# Insulin like growth factor binding protein 7 (IGFBP7) expression is linked to poor prognosis but may protect from bone disease in multiple myeloma

**DOI:** 10.1186/s13045-014-0105-1

**Published:** 2015-02-08

**Authors:** Arnold Bolomsky, Dirk Hose, Martin Schreder, Anja Seckinger, Susanne Lipp, Bernard Klein, Daniel Heintel, Heinz Ludwig, Niklas Zojer

**Affiliations:** Wilhelminen Cancer Research Institute, Department of Internal Medicine I, Wilhelminenspital, Montleartstraße 37, 1160 Vienna, Austria; Medizinische Klinik V, Universitaetsklinikum Heidelberg, Heidelberg, Germany; INSERM U1040, Institute for Research in Biotherapy, CHU Montpellier, Hospital St Eloi, Montpellier, France

**Keywords:** Multiple myeloma, IGFBP7, Microenvironment, Myeloma bone disease, Survival

## Abstract

**Background:**

Insulin like growth factor binding protein 7 (IGFBP7) is a secreted protein binding insulin like growth factor 1 (IGF-1), insulin, vascular endothelial growth factor A (VEGFA), and activin A. It antagonizes bone morphogenetic proteins and is involved in the tumour propagation of solid as well as haematological malignancies. Its role in multiple myeloma (MM) is not defined so far. We therefore aim here to investigate its prognostic and pathophysiological role in MM.

**Methods:**

The clinical significance of *IGFBP7* gene expression was investigated by gene expression profiling in two independent cohorts (n = 948) of newly-diagnosed MM patients. Methylation of the *IGFBP7* promoter was analysed by pyrosequencing and treatment of MM cell lines with 5-aza-2-deoxycytidine. The impact of IGFBP7 on MM cells was studied by CCK-8 assay, BrdU assay and flow cytometry, respectively. *IGFBP7* expression in bone marrow stromal cells (BMSCs) was studied by quantitative RT-PCR. For osteoblast development, immortalized and primary human BMSCs were cultured in osteogenic differentiation medium for 7–14 days in the presence of recombinant human IGFBP7 and/or activin A.

**Results:**

Median *IGFBP7* expression is significantly lower in CD138-purified plasma cells from individuals with MGUS and MM, compared to normal bone marrow plasma cells. *IGFBP7* gene expression in MM cells is regulated by methylation, shown by pyrosequencing and exposure to demethylating agents (5-aza-2-deoxycytidine). High expression of *IGFBP7* in MM cells is associated with adverse survival in two independent cohorts of 247 and 701 newly-diagnosed MM patients treated with high-dose therapy and autologous stem cell transplantation. *IGFBP7* is associated with prognostically adverse chromosomal aberrations (t(4;14) and gain of 1q21), MMSET expression, and higher myeloma cell proliferation. *In vitro*, IGFBP7 overcomes activin A induced osteoblast suppression and promotes osteogenesis. MM cells downregulate IGFBP7 in stromal cells, possibly contributing to the osteoblast suppression found in MM. Conversely, higher *IGFBP7* expression is associated with a lower probability of myeloma bone disease.

**Conclusions:**

Our data indicate that *IGFBP7* expression is a marker for a specific methylation pattern in myeloma, linked to translocation t(4;14) associated MMSET expression, showing clinical features of adverse prognosis with absence of myeloma bone disease.

**Electronic supplementary material:**

The online version of this article (doi:10.1186/s13045-014-0105-1) contains supplementary material, which is available to authorized users.

## Background

Multiple myeloma (MM) is characterized by the accumulation of malignant plasma cells (PCs) in the bone marrow (BM) [1]. MM cells reside in the BM microenvironment similar to their normal counterparts where they receive essential survival and growth signals from the surrounding stromal compartments [2]. Direct interactions between myeloma cells and bone marrow stromal cells (BMSCs) or osteoclasts [3] as well as the crosstalk through various soluble factors play a major role in the pathophysiology of MM leading to clinical features such as myeloma bone disease, anaemia and immunosuppression [1].

MM cells are closely connected with BMSCs, modify their function and induce the production of tumour promoting factors [4]. Within the growth signalling network of the myeloma microenvironment, IL-6, IGF-1 and insulin play a prominent role [5-7]. An imbalance of the RANKL to OPG ratio promotes osteoclast activation [8] while the secretion of osteoblast inhibitory molecules such as DKK-1 and activin A impairs osteogenesis, leading to myeloma induced bone disease [9,10].

Bone morphogenetic proteins (BMPs) in turn stimulate bone formation [11] and are at the same time potent inhibitors of MM cell survival [12,13]. Higher BMP expression in malignant plasma cells is associated with superior overall survival [13]. Less is known, however, about the role of BMP antagonists in MM. In solid malignancies, upregulation of BMP antagonists such as gremlin was shown to promote the growth and survival of tumour cells [14]. We therefore speculated that deregulation of BMP antagonistic proteins in the myeloma microenvironment might be involved in the pathophysiology of MM. To address this question, we analysed the BMP antagonist expression profile in myeloma, identifying insulin like growth factor binding protein 7 (IGFBP7) as a potential gene of interest. IGFBP7 is a secreted protein with IGF-1, insulin [15], VEGFA [16] and activin A [17] binding properties and has been described as BMP antagonist [18]. Methylation dependent silencing of IGFBP7 was reported as associated with unfavourable outcome in lung-, breast-, and pancreatic cancer, as well as hepatocellular carcinoma [19-23]. On the contrary, high *IGFBP7* expression was linked to poor prognosis in oesophageal adenocarcinoma as well as head and neck squamous cell carcinomas [24,25]. Recent studies also suggested a role for IGFBP7 in haematological malignancies. In acute lymphoblastic leukemia (ALL) *IGFBP7* expression was associated with adverse outcome and shown to interfere with leukemia cell proliferation [26,27]. IGFBP7 was also reported to be involved in the crosstalk between BMSCs and ALL cells, mediating asparaginase-resistance in B-lineage ALL cells [27]. Based on these results we were interested in the prognostic and pathophysiologic role of IGFBP7 in MM.

## Results

### *IGFBP7* gene expression is downregulated in myeloma cells

**Figure 1:**
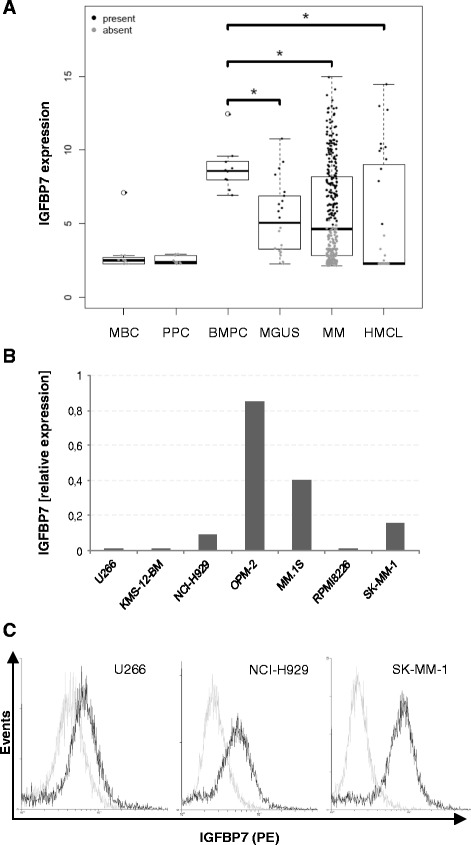
**Insulin like growth factor binding protein 7 (IGFBP7) is downregulated in multiple myeloma. (A)** IGFBP7 expression levels were analysed by gene expression profiling of memory B cells (MBC), polyclonal plasmablastic cells (PPC) as well as of CD138+ purified cells from healthy donors (BMPC), newly diagnosed myeloma patients (MM), patients suffering from monoclonal gammopathy of undetermined significance (MGUS) and human MM cell lines (HMCLs). Grey data points indicate an absent, black data points a present Affymetrix detection call. The asterisk indicates statistical significance (* *P* < 0.02). IGFBP7 expression analysis by qPCR **(B)** and flow cytometry **(C)** confirmed heterogeneous expression levels in myeloma cell lines. **(B)** Expression levels by qPCR are displayed relative to peripheral blood mononuclear cells (PBMCs) of healthy donors (n = 3). *IGFBP7* expression in PBMCs was arbitrarily set at 1. **(C)** Intracellular staining of MM cells for IGFBP7 protein levels confirmed the variable expression of IGFBP7 transcripts observed by qPCR. MM cells were stained with either anti-IGFBP7 antibody (black lines) or control antibody (light grey lines), followed by PE-conjugated anti-goat antibody.

*IGFBP7* gene expression levels were significantly decreased in a series (HM group) of CD138 sorted plasma cells from MGUS (n = 22) and MM patient samples (n = 332) as well as in human myeloma cell lines (HMCLs) (n = 32) compared to normal plasma cells (n = 10) (*P* < 0.02) whereas *IGFBP7* expression was absent in memory B cells (MBCs) and proliferating polyclonal plasmablastic cells (PPCs) (Figure 1A). *IGFBP7* gene expression was detectable in 100% of purified bone marrow plasma cells from healthy individuals, compared to 45.5% of samples from MGUS patients, 47.7% from MM patients, 47.1% of HMCLs and 0% of MBCs and PPCs, respectively. Expression levels varied widely in bone marrow plasma cell samples from myeloma patients as well as in HMCLs, the latter confirmed by PCR analysis (Figure 1B) and flow cytometry (Figure 1C). Other factors related to BMP antagonism dysregulated in MM are listed in Additional file 1: Table S1 and Additional file 2: Table S2.

### *IGFBP7* gene expression is regulated via methylation

**Figure 2:**
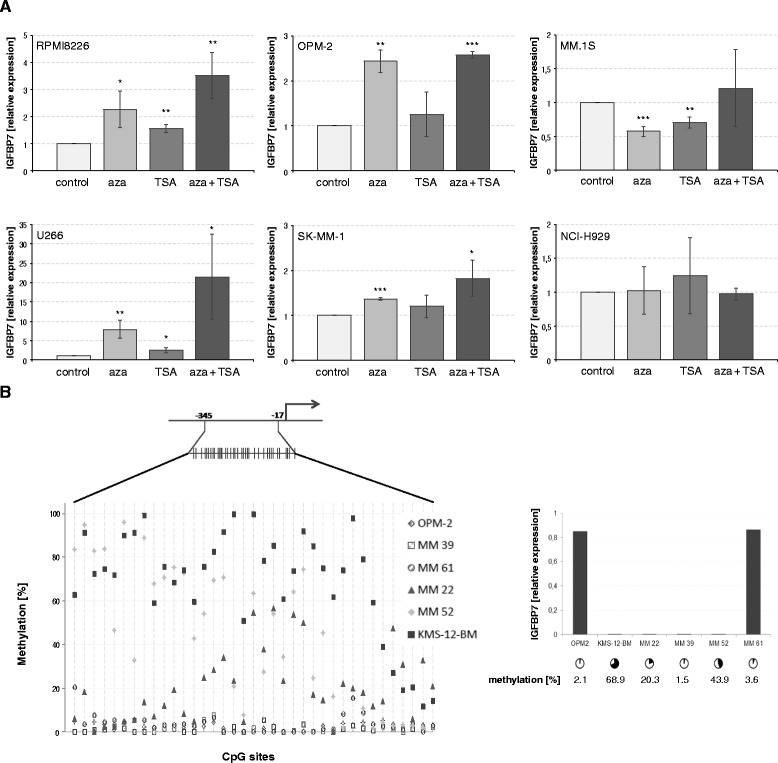
**IGFBP7 is silenced via methylation. (A)** MM cell lines were treated with DMSO (0.1%) or 5-aza-2′deoxycytidine (500 nM) for 96 hours. Trichostatin A (50 ng/ml) was added for the last 24 hours of incubation. Subsequently *IGFBP7* expression was analysed by quantitative PCR. Values represent the mean ± SD of three independent experiments performed in triplicates. Asterisks indicate statistical significance compared with the control (* *P* < 0.05, ** *P* < 0.01, *** *P* < 0.001). **(B)** Pyrosequencing was performed along 328 base pairs within the *IGFBP7* promoter region containing 37 CpG sites (schematic representation). The graph on the left displays the percentage of methylation of each individual CpG site for all samples analysed. The graph on the right represents the corresponding *IGFBP7* expression values in the samples used for pyrosequencing. Cycles indicate the mean percentage of DNA methylation along all 37 CpG sites in the promoter region analysed.

Exposure of HMCLs to the demethylating agent 5-aza-2′ deoxycytidine (aza) and/or the histone deacetylase inhibitor Trichostatin A (TSA) showed significant upregulation of *IGFBP7* mRNA levels in 4 of 6 cell lines tested (median upregulation with aza + TSA: 3.05; range: 1.83 – 21.47; *P* < 0.05) (Figure 2A). In addition, in KMS-12-BM, *IGFBP7* expression changed from not detectable by qPCR in the DMSO control to detectable with aza treatment (not shown). To validate these results, we analysed the promoter methylation status of *IGFBP7* in a “low” (KMS-12-BM) and “high” (OPM-2) expressing MM cell line as well as in CD138 purified cells of four myeloma patients. Pyrosequencing demonstrated methylation of the *IGFBP7* promoter region generally in accordance with the *IGFBP7* gene expression levels by qPCR (Figure 2B). The methylation status of one patient, however, was not in line with the qPCR results, showing no methylation in the analysed promoter region despite suppressed *IGFBP7* expression. This suggests that methylation of other regulatory regions or other mechanisms might mediate downregulation of *IGFBP7* gene expression in certain MM cases.

### *IGFBP7* gene expression in MM cells of patients is linked to adverse survival and poor risk cytogenetics

**Figure 3:**
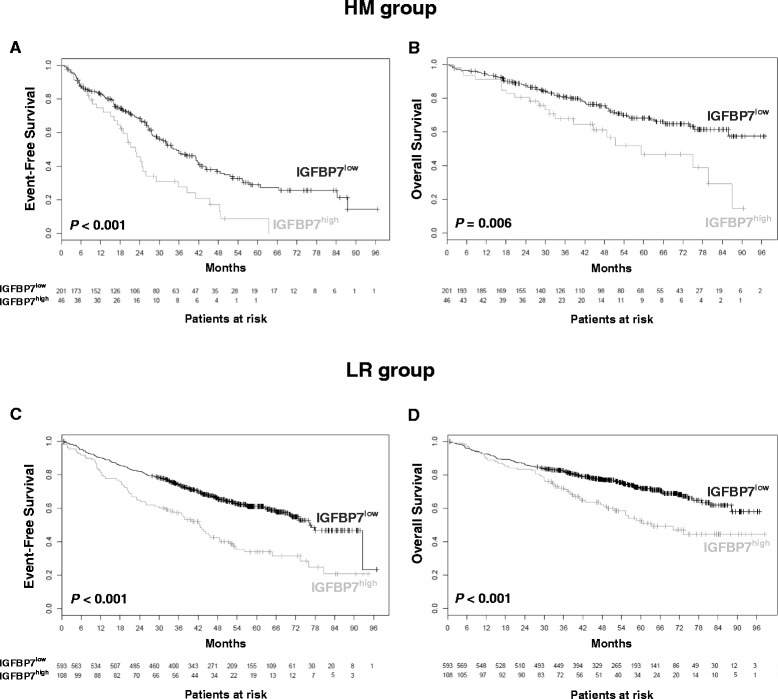
**High IGFBP7 expression is predictive for poor outcome.** *IGFBP7* expression was analysed in CD138+ purified MM cells of newly diagnosed, previously untreated myeloma patients of the Heidelberg-Montpellier group (HM; n = 247), as well as for validation purposes in the independent Little-Rock (LR; n = 701) patient cohort as depicted in the Methods section. In both patient series, high *IGFBP7* expression was associated with worse event-free **(A, C)** and overall survival **(B, D)**.

*IGFBP7* gene expression in MM cells was significantly associated with event free survival (EFS) and overall survival (OS) in both patient cohorts analysed. Patients with high *IGFBP7* expression levels had a significantly worse outcome compared to those with low *IGFBP7* expression in the HM (median EFS: 34.2 vs. 22.6 months, *P* < 0.001; median OS: NA vs. 59.1 months, *P* = 0.006) and the LR group (median EFS: 76.4 vs. 42.5 months, *P* < 0.001; median OS: NA vs. 63.3 months, *P* < 0.001) (Figure 3). As optimal cut-offs for *IGFBP7* expression were different between the cohorts (see methods section), we applied the same fraction of patients defined as “high” IGFBP7 expressers in the HM-cohort (46/274, 18.6%) to the LR-cohort (131/701, 18.6%), confirming the association with EFS and OS (Additional file 3: Figure S1).

High expression of *IGFBP7* was likewise associated with poor risk cytogenetics, most notably translocation t(4;14)(*P* < 0.0001) and gain of 1q21 (*P* = 0.039). No significant correlation was observed with the translocation t(11;14) and deletion 17p (Additional file 4: Figure S2). The association between *IGFBP7* expression levels and t(4;14) was further validated by an *in-silico* analysis of two additional publically available GEP-datasets [28,29]. In both, *IGFBP7* expression was significantly higher in the GEP-defined MMSET^high^ expressing (typically t(4;14) positive) patients compared to the other molecular subgroups (Additional file 5: Figure S3).

High *IGFBP7* expression levels were also associated with the GEP-based proliferation index (GPI) for the HM (*R* = 0.21, *P* = 0.008) and LR group (*R* = 0.22, *P* = 0.037), which in turn is associated with adverse survival. *IGFBP7* expression was not associated with tumour mass surrogates such as ISS stage or beta 2 microglobulin (B2M) levels.

### IGFBP7 impacts on myeloma cell proliferation and viability

**Figure 4:**
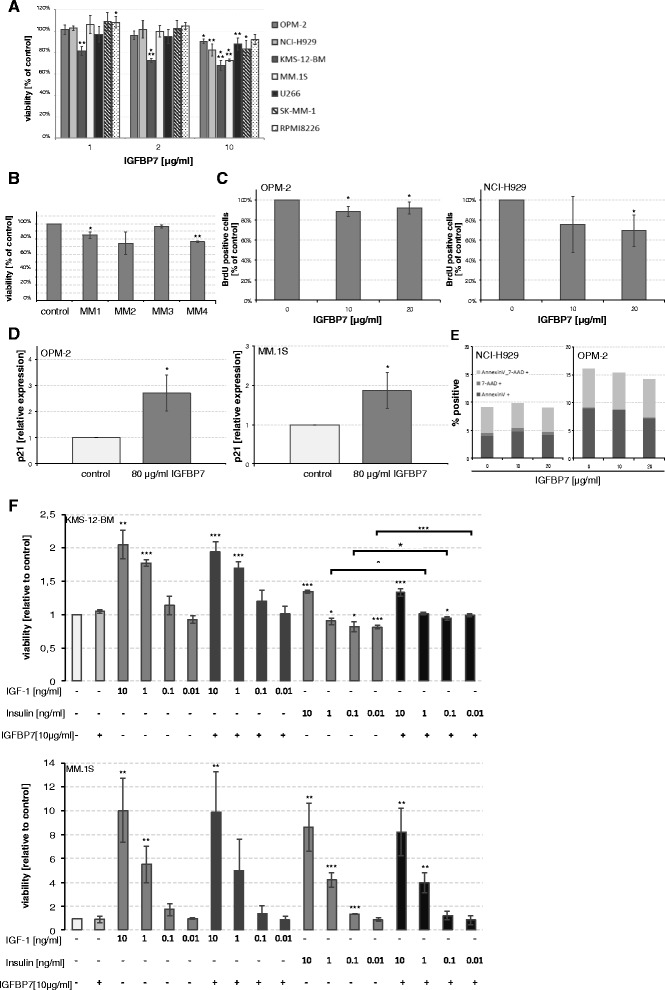
**IGFBP7 inhibits myeloma cell proliferation.** MM cell lines **(A)** and primary MM cells **(B)** were treated with PBS/BSA 0.1% or recombinant human IGFBP7 (rhIGFBP7) at the indicated concentrations for 96 hours. Primary cells were treated with IGFBP7 at 10 μg/ml. Viable cell numbers were evaluated as outlined in the methods section. **(C)** Proliferation of MM cells was assessed by BrdU assay. HMCLs were treated for 72 hours with PBS/BSA 0.1% or rhIGFBP7 at the indicated concentrations. BrdU was added for the last 19 hours of incubation. Proliferation was assessed by measuring absorbance at 450 nm. **(D)** Expression of p21 was analysed by quantitative PCR after 24 hour treatment with PBS/BSA 0.1% or rhIGFBP7 at 80 μg/ml. **(E)** Apoptosis of HMCLs was determined by Annexin V and 7-AAD staining after a 72 hour incubation period in the presence of PBS/BSA 0.1% or rhIGFBP7 at the indicated concentrations. Graphs represent the mean (% positive cells on FACS) of three independent experiments performed in duplicates. **(F)** KMS-12-BM and MM.1S were cultured in Syn-H serum free medium in the presence of rhIGFBP7, IGF-1 and/or insulin at the indicated concentrations for 96 hours before viable cell numbers were determined. **(A-D, F)** Values represent the mean ± SD of three independent experiments performed in triplicates and mean ± SD of triplicate experiments for primary MM cells. Asterisks indicate statistical significance compared with the control (* *P* < 0.05, ** *P* < 0.01, *** *P* < 0.001).

Exposure to recombinant human IGFBP7 (rhIGFBP7) weakly but significantly reduced viable cell numbers in 6 of 7 HMCLs in a dose dependent manner (relative viability compared to control: 0.68 ± 0.05 - 0.91 ± 0.02, *P* < 0.05). In RPMI8226, treatment with rhIGFBP7 at 10 μg/ml similarly led to a minor reduction of viable cell numbers by 7.8 ± 4.9% compared to control, although not statistically significant (*P* = 0.05)(Figure 4A). In line with this, treatment with rhIGFBP7 slightly reduced viability in 4 of 4 primary MM cell samples tested, reaching statistical significance in 2 of 4 samples (Figure 4B). Analysis of proliferation by BrdU assay mirrored the viability assessment, showing weakly reduced proliferation of MM cells exposed to IGFBP7 (BrdU positive cells relative to control: 0.92 ± 0.04 and 0.69 ± 0.16 with 20 μg/ml rhIGFBP7 in OPM-2 and NCI-H929, respectively; *P* < 0.05) (Figure 4C). Analysis of cell cycle inhibitory proteins after short-term treatment with rhIGFBP7 showed a significant upregulation of p21 in OPM-2 and MM.1S (relative induction 2.7 ± 0.68 and 1.9 ± 0.45 compared to control, respectively; *P* < 0.05) (Figure 4D), while no significant changes were observed in the expression levels of p16 and p27 (data not shown). In contrast, no induction of apoptosis in MM cells could be observed related to IGFBP7 exposure (Figure 4E).

As IGFBP7 harbors binding sites for IGF-1 and insulin we also examined whether IGFBP7 alters the stimulatory activity of these growth factors. IGF-1 and insulin at 0.01-10 ng/ml potently stimulated the survival in 2 of 4 MM cell lines in Syn-H serum free medium (IGF-1 10 ng/ml: 2.1 ± 0.2 and 10.0 ± 2.7 fold viable cells compared to control in KMS-12-BM and MM.1S, respectively; insulin 10 ng/ml: 1.3 ± 0.02 and 8.6 ± 2.0), which was not abrogated by combined treatment with 10 μg/ml rhIGFBP7 (Figure 4F).

### MM cells induce downregulation of IGFBP7 in BMSCs

We next characterized the expression of *IGFBP7* in BM stromal cells. *IGFBP7* expression was 31 times higher in BMSC TERT+ and 11 fold higher in primary (normal) BMSCs compared to peripheral blood mononuclear cells. In comparison with OMP-2, the highest expressing MM cell line in our analysis, *IGFBP7* expression was 37 fold higher in BMSC TERT+ cells and 13 fold higher in primary BMSCs.

**Figure 5:**
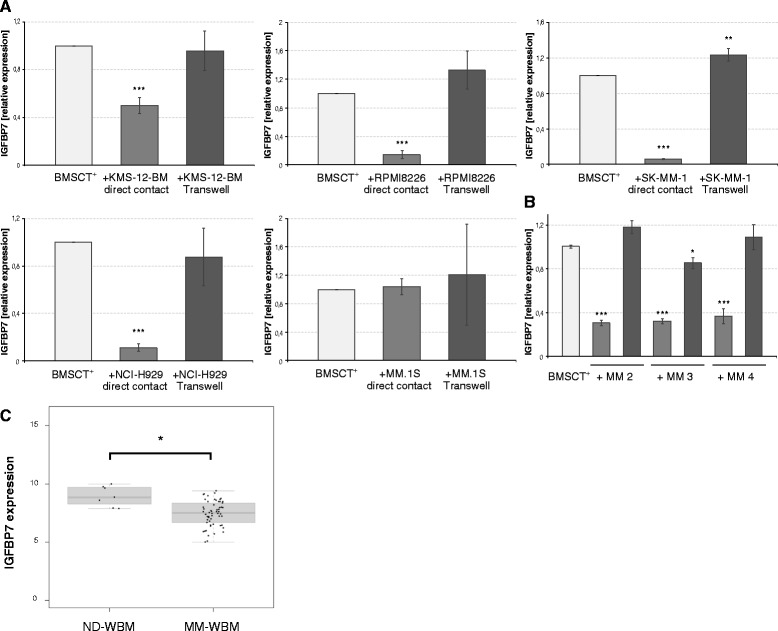
**Myeloma cells downregulate IGFBP7 in BMSCs.** Co-culture of hBMSC TERT+ cells and MM cell lines **(A)** or primary MM cells **(B)** was performed in the presence or absence of transwell inserts for 72 hours. Human BMSC TERT+ monocultures served as control. Direct contact cultures were separated by MACS sorting previous to RNA isolation of the CD138-negative fraction. *IGFBP7* mRNA levels were analysed by quantitative PCR and compared to the control. Values represent the mean ± SD of three independent experiments performed in triplicates for MM cell lines and mean ± SD of triplicate experiments for primary MM cells. **(C)** Downregulation of *IGFBP7* in the myeloma microenvironment was confirmed by GEP analysis of whole bone marrow (WBM) samples obtained from healthy donors (ND) and newly diagnosed myeloma patients (MM). Asterisks indicate statistical significance compared with the control (* *P* < 0.05, ** *P* < 0.01, *** *P* < 0.001).

Co-cultures of BMSC TERT+ cells and HMCLs showed a significant downregulation of *IGFBP7* in BMSCs in the presence of 4 of 5 HMCLs in a contact dependent manner (relative expression compared to control: 0.06 - 0.50; *P* < 0.05) (Figure 5A). Similar results were obtained using primary MM cell samples (*P* < 0.001) (Figure 5B). Thus, MM cells have the capacity to significantly downregulate the expression of *IGFBP7* in BM stromal cells, which highly express *IGFBP7* in monoculture. When *IGFBP7* expression levels in whole BM samples of myeloma patients were compared with those of healthy donors, significantly lower levels in the MM samples were likewise found (*P* = 0.012) (Figure 5C).

### IGFBP7 promotes osteoblast development and might protect from myeloma bone disease

**Figure 6:**
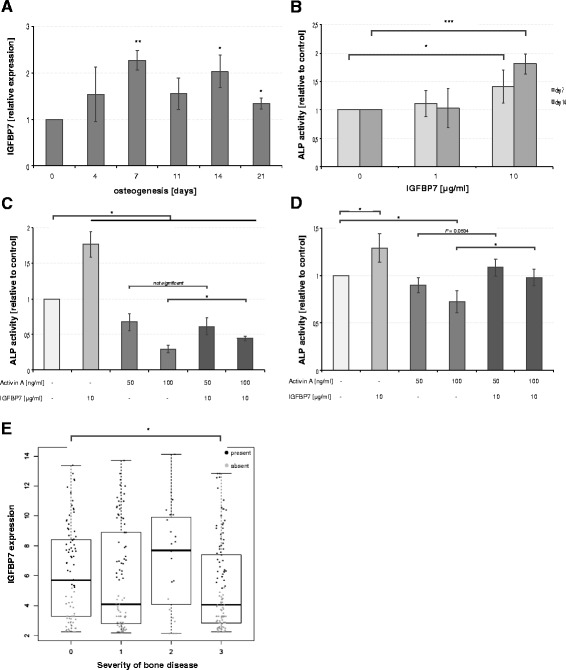
**IGFBP7 promotes osteogenesis and is associated with myeloma bone disease. (A)** Primary BMSCs were cultured in osteogenic medium for 21 days. *IGFBP7* expression was analysed during osteogenesis by quantitative PCR at the indicated time points. The graph represents the relative expression compared to day 0 of osteogenesis. **(B)** Alkaline phosphatase activity in primary BMSCs was determined at days 7 and 14 of differentiation in the presence or absence of rhIGFBP7 (1 or 10 μg/ml) and is displayed relative to the control (PBS/BSA 0.1%). For neutralization experiments, alkaline phosphatase activity in primary human BMSCs **(C)** and BMSC TERT^+^ cells **(D)** was analysed at day 14 of osteogenesis in the presence of activin A (50 or 100 ng/ml) and/or rhIGFBP7 (10 μg/ml). Results are displayed relative to the control (PBS/BSA 0.1%). **(A-D)** Values represent the mean ± SD of three independent experiments performed in triplicates. **(E)** *IGFBP7* expression was analysed with regard to the severity of myeloma bone disease. Grey data points indicate an absent, black data points a present Affymetrix detection call. Asterisks indicate statistical significance compared with the control (* *P* < 0.05, ** *P* < 0.01, *** *P* < 0.001).

As BMSCs have multilineage potential and can differentiate into osteoblasts, we were interested in the role of IGFBP7 in osteoblast development. *IGFBP7* gene expression increased during the course of differentiation of primary (ND) BMSCs (Figure 6A) and treatment with rhIGFBP7 further stimulated osteoblast formation, indicated by a dose dependent increase in alkaline phosphatase activity at day 7 (1.42 ± 0.29 fold increase compared to control; *P* < 0.05) and 14 (1.81 ± 0.18 fold increase compared to control, *P* < 0.001 ) of osteogenesis (Figure 6B). Interestingly, we also observed a significant reduction in Dickkopf-1 expression with rhIGFBP7 treatment during osteogenesis (0.22 ± 0.06 fold decrease compared to control; *P* < 0.05) but no change in other OB marker genes such as Runx2, Col1A and Dlx5 (data not shown).

As IGFBP7 was shown to bind activin A [17] we studied whether rhIGFBP7 treatment could overcome the inhibitory action of activin A on osteogenesis. As expected, activin A demonstrated osteoblast inhibitory properties reflected by a dose dependent decrease of alkaline phosphatase activity. Importantly, treatment with rhIGFBP7 was able to partially reverse this effect in primary and immortalized BMSCs (Figure 6C-D). In our patient series we observed significantly lower expression levels of *IGFBP7* in MM cells of patients with advanced bone disease compared to patients with no osteolytic lesions (Figure 6E).

## Discussion

Various soluble factors have been identified in the signaling crosstalk between myeloma and stromal cells including IGF-1, IL6, DKK-1, RANKL and activin A [1,2,5-10]. In this study we identified IGFBP7, a secreted factor with BMP antagonistic activity [18], and binding sites for IGF-1, insulin [15], VEGFA [16] and activin A [17] as a potential novel player in the pathogenesis of MM, including myeloma bone disease. BMPs are known to induce apoptosis in MM cells [12,13], so upregulation of BMP antagonists might be envisaged to be used as a protective mechanism by myeloma cells. Median *IGFBP7* expression levels, although *IGFBP7* displayed a heterogenous expression profile, were found to be downregulated in malignant plasma cells compared to their normal counterparts, suggesting that myeloma cells do not rely on the BMP antagonistic activity of IGFBP7.

We show that *IGFBP7* downregulation in MM cells can be explained by promoter methylation. Such a downregulation of *IGFBP7* by promoter methylation was previously observed in solid tumours including melanoma, prostate, lung and breast cancer as well as T-ALL, suggesting a common mechanism in cancer [19,21,30-32]. Plasma cells from MGUS patients already display reduced *IGFBP7* expression, indicating that deregulation of IGFBP7 might occur early in the course of the disease.

IGFBP7 belongs to the family of insulin like growth factor binding proteins (IGFBPs) consisting of six high- and several low-affinity members [33]. IGFBPs were described to act via IGF dependent and independent mechanisms [33]. Contrary to other family members, in particular IGFBP3, IGFBP7 possesses low binding affinity for IGFs [15,34]. Accordingly, unlike IGFBP3 [6], IGFBP7 was not able to antagonize the growth stimulatory activity of IGF-1 and insulin on MM cell lines.

Previous studies indicated a role for IGFBP7 as tumour suppressor in several solid malignancies including melanoma, hepatocellular carcinoma, thyroid, breast, bladder and colon cancer by either direct induction of apoptosis and/or inhibition of tumour growth by deregulation of p16, p21, p53 and ERK signaling [19,26,35-38]. However, results regarding the role of IGFBP7 in solid tumours are inconsistent and recent studies provide a more complex picture of IGFBP7 in different entities. In glioblastoma, IGFBP7 was shown to stimulate tumour cell proliferation and angiogenesis of brain endothelial cells [39,40]. Similarly, elevated *IGFBP7* expression was detected in specific stages of colorectal cancer and silencing of IGFBP7 reduced proliferation as well as colony formation in colorectal cancer cell lines [41]. Expression of *IGFBP7* was also detected in cancer-associated fibroblasts, endothelial cells, mesenchymal tumours and malignant epithelial cells with a mesenchymal phenotype. In the latter, loss of IGFBP7 significantly impaired the anchorage independent growth of tumour cells. Furthermore, *IGFBP7* expression in cancer stromal cells supported the growth of colon cancer cells [41]. These findings establish a complex variety of functions for IGFBP7 depending on the malignancy investigated.

In haematological malignancies, IGFBP7 was found to be associated with BAALC expression in T-ALL and to correlate with poor survival [26]. Similarly, in B-cell ALL *IGFBP7* expression in BMSCs was found to be associated with asparaginase resistance and decreased leukemia-free survival [27]. In our study, high expression levels of *IGFBP7* predicted poor outcome in two large and independent cohorts of MM patients. Whereas the mean values and standard deviations (5.564 ± 3.213 vs. 3.155 ± 1.966) as well as the percentage of patients expressing *IGFBP7* as defined by the PANP algorithm (marginal and present, 44.9 vs. 12.9%) significantly differed between the HM- and LR-cohort, the prognostic impact of *IGFBP7* expression was comparable between both cohorts. Observed differences in frequency and height of expression are most likely due to different amplification protocols used (double vs. single amplification) [42-44].

*IGFBP7* expression in MM cells was linked to prognostically adverse chromosomal aberrations such as translocation t(4;14) and amplification 1q21. The multiple myeloma SET domain (MMSET) protein, specifically overexpressed in t(4;14) myeloma, is a histone methyl transferase shown to modulate DNA methylation, thereby inducing the activation of specific target genes [45]. Interestingly, *IGFBP7* expression was found to be regulated by MMSET in myeloma cells [45] which is in line with our observation of higher IGFBP7 transcript levels in t(4;14) cases. The association between MMSET expression and expression of *IGFBP7* was confirmed by *in-silico* analysis of two open-source GEP datasets. This suggests that IGFBP7 represents a novel marker of high-risk myeloma, defined by an epigenetic signature and regulated by MMSET.

The growth attenuating effects of IGFBP7 we observed *in vitro* were rather small and evident only at high IGFBP7 concentrations [46]. Moreover, we show that, in clinical myeloma samples, *IGFBP7* expression is associated with higher myeloma cell proliferation, in turn associated with adverse prognosis. The phenotype of MMSET expressing myeloma seems to overrule the weak inhibitory activity of IGFBP7 on myeloma cell proliferation. Thus, rather than being mechanistically involved in the poor outcome, IGFBP7 might rather be seen as a marker associated with a high-risk disease phenotype.

We show upregulation of IGFBP7 in BMSCs while undergoing osteogenic differentiation and treatment with exogenous rhIGFBP7 further stimulating osteoblast activity. IGFBP7 treatment led to a significant downregulation of DKK1, a major osteoblast inhibitory molecule in MM [9]. Furthermore, we were able to show that IGFBP7 can neutralize the inhibitory effects of activin A on osteoblast development. Activin A mediated inhibition of osteogenesis leads to uncoupling of the delicate balance between bone formation and degradation thus contributing to myeloma related bone destruction [10,47]. Our results suggest that the downregulation of IGFBP7 in the myeloma microenvironment enables activin A to release its full destructive potential.

Possibly, the lack of IGFBP7 in the microenvironment is aggravated by low or absent intrinsic expression of *IGFBP7* in myeloma cells. In contrast, in myeloma cases with high *IGFBP7* expression the downregulation of this molecule in the microenvironment might be counterbalanced by production in myeloma cells, possibly explaining the correlation of higher intrinsic *IGFBP7* expression and absence of advanced bone disease. In line with this, prior studies reported osteolytic lesions to occur less frequently in myeloma cases harboring a translocation t(4;14) [48].

## Conclusions

Taken together, our results suggest that IGFBP7 might play a dual role in the pathophysiology of MM. Maintained expression of *IGFBP7* in myeloma cells represents a novel prognostic marker linked to prognostically adverse chromosomal aberrations and a specific epigenetic profile. In stromal cells in the vicinity of MM cells *IGFBP7* expression is suppressed, releasing the full potential of osteoblast inhibitory molecules like activin A, which then promote myeloma bone disease. In addition, these findings might provide a mechanistic link for the reduced frequency of osteolytic lesions in t(4;14) associated high-risk myeloma.

## Methods

### Patients

Patients presenting with previously untreated MM (n = 332) or monoclonal gammopathy of unknown significance (MGUS; n = 22) at the University Hospitals of Heidelberg and Montpellier (HM group) as well as 10 healthy normal donors have been included in the study approved by the Heidelberg ethics committee (#229/2003 and S-152/2010) after written informed consent was obtained in accordance with the Declaration of Helsinki. Patients were diagnosed, staged and response to treatment assessed according to standard criteria. Normal bone marrow plasma cells and myeloma cells were purified as previously published [13,44]. Memory B cells (MBCs; n = 11) and polyclonal plasmablasts (PPCs; n = 10) were obtained as reported [49]. Aliquots of unpurified whole bone marrow from myeloma patients (n = 154) and healthy donors were obtained after NH_4_ lysis.

Bone disease was assessed by conventional X-ray and whole body CT scan and graded as 0 (normal bone structure), 1 (osteopenia/osteoporosis), 2 (1–3 osteolyses) or 3 (major structural damage, >3 osteolyses) as described previously [44].

The prognostic impact of *IGFBP7* expression data was validated on an independent cohort of 701 newly diagnosed, therapy-requiring patients treated within the total therapy 2 or 3 protocol, respectively (LR group), for whom gene expression data are publicly available (see below) [50,51]. All patients in both cohorts, i.e. the HM- and the LR-group, received high-dose chemotherapy followed by autologous stem cell transplantation.

### Cells and culture conditions

Human multiple myeloma cell lines (HMCLs) U266, KMS-12-BM, OPM-2, NCI-H929, SK-MM-1 and RPMI8226 were obtained from the German Collection of Microorganisms and Cell Cultures (Braunschweig, Germany). MM.1S cells were kindly provided by Dr Steven Rosen (Northwestern University, Chicago, IL). All HMCLs were cultivated in RPMI-1640 medium supplemented with 10% heat-inactivated fetal bovine serum, 2 mM L-glutamine and 100 U/ml penicillin/streptomycin (Gibco).

A human bone marrow mesenchymal stromal cell line immortalized by enforced expression of telomerase (hBMSC TERT+) was kindly provided by Dr Dario Campana (St. Jude Children’s Research Hospital, Memphis, TN) and cultured in RPMI-1640 medium supplemented with 10% FBS, 2 mM L-glutamine, 100 U/ml penicillin/streptomycin and 1 μM hydrocortisone (Sigma-Aldrich). Primary human BMSCs (Lonza) were cultured in α-MEM (Gibco) supplemented with 10% FBS, 2 mM L-Glutamine, 100 U/ml penicillin/streptomycin and 1 ng/ml FGF-2 (Peprotech).

For co-culture experiments, 0.25 × 10^6^ TERT^+^ hBMSCs were seeded in 6-well plates and cultured over night before 0.5 × 10^6^ MM cells were added per well for 72 hours in the presence or absence of transwell inserts (0.4 μm pore size; BD). Direct contact cultures were MACS sorted by CD138 selection previous to RNA isolation of the CD138-negative population. Purity of the negative sort was ≥ 90% by cytological assessment.

### Cytotoxicity assay

Cell Counting Kit-8 (Sigma) was used to analyze the impact of recombinant human IGFBP7 (rhIGFBP7; Peprotech) on myeloma cell growth following the manufacturer’s directions. In brief, 2 × 10^4^ cells per well were seeded in flat-bottomed 96-well plates (Iwaki) in the presence of rhIGFBP7 (0–10 μg/ml). Neutralization experiments with recombinant human IGF-1 (10 or 100 ng/ml) (R&D Systems), insulin (10 or 100 ng/ml) (Roche Diagnostics) and IGFBP7 (1 or 10 μg/ml) were performed in serum free Syn-H medium (ABCell-Bio) according to Sprynski *et al*. [6]

### BrdU assay

Proliferation of myeloma cells was assessed by BrdU assay (Calbiochem) following the manual. BrdU label was added for the last 19 hours of the culture period. Proliferation was determined by absorbance measurement at 450 nm using a HTS 7000 Bio Assay Reader (Perkin Elmer).

### Flow cytometry

Intracellular staining of IGFBP7 was performed using the BD Cytofix/Cytoperm™ Plus Kit (BD Biosciences) according to the manual. After fixation and permeabilization, cells were incubated with the primary goat-anti-human IGFBP7 antibody (sc-6064; Santa Cruz Biotechnology Inc., Santa Cruz, CA, USA) or the corresponding isotype control (sc-3887; Santa Cruz) for 30 min at 4°C. Thereafter cells were washed and incubated for 30 min at 4°C with a secondary PE-conjugated donkey-anti-goat antibody (sc-3857; Santa Cruz). Analysis was performed on a FACScan (BD Biosciences).

Induction of apoptosis was determined by FACS analysis of Annexin V/7-AAD stainings (BD Biosciences). HMCLs were seeded at a concentration of 2.5 ×10^5^/ml and treated with either PBS/BSA 0.1% (control) or rhIGFBP7 (10–20 μg/ml) for 72 hours. Cells were incubated for 15 min with Annexin V and 7-AAD in the dark before performing analysis.

### Quantitative RT-PCR

Total RNA was isolated using RNeasy kit (Qiagen) and cDNA synthesis was performed with M-MuLV reverse transcriptase (New England Biolabs). IGFBP7, p16, p21, p27, Runx2, Dlx-5, Col1A and DKK-1 expression levels were analysed by quantitative PCR (qPCR) using TaqMan Universal PCR Master Mix and pre-designed TaqMan gene expression assays (Applied Biosystems). RPLPO served as endogenous control. Reactions were carried out in 25 μl volumes and run on the ABI Prism 7300 platform (Applied Biosystems). All samples were run at least in duplicates.

### Methylation

For IGFBP7 induction experiments HMCLs were seeded at 2 ×10^5^/ml and cultivated in the presence of 0.1% DMSO (control) or 500 nM 5-aza-2′-deoxcytidine (aza; Sigma) for 96 hours. Trichostatin A (TSA; Sigma) was added at a concentration of 50 ng/ml for the last 24 hours of the culture period.

Analysis of the IGFBP7 promoter methylation status was performed by varionostic GmbH (Ulm, Germany) using pyrosequencing technology.

### Osteogenic differentiation

For osteoblast development, BMSCs were seeded at a density of 12500–25000 per cm^2^ and grown to 70-80% confluence. Osteoblast differentiation was initiated by changing the medium to α-MEM supplemented with 15% FBS, 2 mM L-Glutamine, 100 U/ml penicillin/streptomycin, 10 nM dexamethasone (Sigma), 50 μg/ml ascorbic acid (Sigma) and 10 mM β-glycerophosphate (Sigma). Osteogenic medium was changed every 3–4 days. Recombinant human IGBFP7 (1 or 10 μg/ml) and/or activin A (50 or 100 ng/ml) (Peprotech) were added with every medium change. Cells treated with PBS/BSA 0.1% served as control.

### Alkaline phosphatase activity assay

Alkaline phosphatase activity was determined at day 14 of differentiation using the AttoPhos® AP Fluorescent Substrate System (Promega) following the manual. Fluorescence was measured at 595 nm with excitation at 430 nm. A standard curve was obtained by diluting the supplied Calibrator solution (500 ng BBT/ml) in AttoPhos buffer.

### Gene expression profiling

Gene expression profiling was performed as previously published [44] using U133 2.0 plus arrays according to the manufacturer’s instructions (Affymetrix, Santa Clara, CA, USA). Expression data are deposited in Array Express under the accession numbers E-MTAB-317 as well as E-TABM-1138 and Gene Expression Omnibus GSE24080 (the latter two for the LR group). When different probe sets were available for the same gene, we chose the probe set yielding the maximal variance and the highest signal.

### Interphase fluorescence in situ hybridization

Analyses were performed on CD138-purified plasma cells as described [52] using probes for the detection of numerical aberration of the chromosomal regions 1q21, 5p15/5q35, 9q34, 13q14.3, 15q22, 17p13, as well as the IgH-translocations t(11;14)(q13;q32), and t(4;14)(p16.3;q32) (Kreatech, Amsterdam, The Netherlands and MetaSystems, Altlussheim, Germany).

### Statistical analysis

Correlations (Spearman and Pearson) and survival analysis (Cox regression analysis) were performed using SPSS statistics 17.0. For the analysis of *in vitro* experiments, two-tailed unpaired *t* test was performed using Prism 5 (GraphPad Software Inc., La Jolla, CA, USA). *P*-values <0.05 were considered to be statistically significant.

Gene expression analyses were performed on GC-RMA preprocessed data sets. To assess presence (“expressed”) or absence (“not expressed”) of gene expression, the “Presence-Absence calls with Negative Probesets” (PANP) algorithm was used [53]. For the analysis of the prognostic value of IGFBP7, patients with high and low *IGFBP7* expression in the HM cohort were delineated using maximally selected rank statistics as implemented in the maxstat R package (http://cran.r-project.org/web/packages/maxstat/index.html). The survival function was assessed by means of the Kaplan-Meier method (rms package in R). The log rank test was used to test for statistically significant survival curve differences. An effect was considered as statistically significant if the *P*-value of its corresponding statistical test was not higher than 5%. All computations were performed using R 3.1.0 (http://www.r-project.org/) and Bioconductor 2.14.

For validation of the impact of *IGFBP7* expression on survival, cel-files from 701 patients treated within the total therapy 2 or 3 protocol (LR group), were preprocessed as described above (GC-RMA), and the same analysis strategy followed to obtain an optimal cut-off (maxstat), i.e. the optimal cut-off was derived as the mean of the cut-offs derived for EFS and OS. This separate analysis yielded two different cut-off values for the HM- and LR-cohort. As the generation of two different cut-offs for the HM and LR-group, i.e. delineating different fractions of patients between in the different cohorts, might give an over-optimistic validation, we likewise applied the fraction of patients defined as “high IGFBP7 expressing” from the HM-cohort (46/247, 18.6%) to the LR-cohort (131/701, 18.6%).

## Additional files

Additional file 1: Table S1. BMP antagonist expression in CD138+ purified cells. Bold indicates differential expression in MM compared to healthy donor bone marrow plasma cells (BMPCs). Additional file 2: Table S2. BMP antagonist expression pattern in whole bone marrow (WBM) samples. Bold indicates differential expression. Additional file 3: Figure S1. EFS and OS in the LR cohort when the same fraction of patients defined as “high” IGFBP7 expressers in the HM-cohort (18.6%) was designated as high expressers in the LR-cohort. High *IGFBP7* expression was associated with adverse event-free (*P*<0.001) and overall survival (*P*<0.002) by applying this criterion. Additional file 4: Figure S2. IGFBP7 expression is associated with high risk cytogenetics. Gene expression analysis of the HM-patient cohort revealed that high *IGFBP7* expression levels were associated with poor risk cytogenetic markers including (A) translocation t(4;14) and (C) amplification 1q21. Moreover, a trend was found regarding the more frequent presence of deletion 13q in patients with high *IGFBP7* expression values (D). No association was observed for (B) translocation t(11;14) and (E) deletion 17p. Grey data points indicate an absent, black data points a present Affymetrix detection call. Additional file 5: Figure S3. IGFBP7 expression is elevated in MMSET defined molecular subgroups of myeloma. IGFBP7 expression was analysed in two publically available GEP datasets published by (A) Zhan *et al*. [33] and (B) Agnelli *et al*. [34]. Both datasets classified MM cases into distinct molecular subgropus based on microarray experiments. (A) IGFBP7 expression was significantly elevated in the MMSET (MS) overexpressing subgroup compared to five other GEP-defined subgroups. In addition, IGFBP7 expression tended to be elevated in the MS subgroup compared to the t(11;14) associated cyclin D1 (CD1) overexpressing samples. The other subgoups were defined by a proliferation (PR), low bone involvement (LB), hyperdiploid (HY), t(6;14) associated cyclin D (CD2) and MAF (MF) expression based GEP profile. (B) Analysis of the dataset from Agnelli *et al*. [34] confirmed overexpression of *IGFBP7* in the MMSET defined molecular subgroup of MM (TC4). The other subgroups were defined by cyclin D overexpression (TC1), hyperdiploid status (TC2) and MAF overexpression (TC5.) TC3 was defined by the lack of association with any of the other subgroups. Asterisks indicate statistical significance compared with the other molecular subgroups (* *P* < 0.05). Horizontal lines represent median *IGFBP7* expression with interquartile range.

## Abbreviations

ALL: acute lymphoblastic leukemia
aza: 5-aza-2′-deoxcytidine
BM: bone marrow
BMP: bone morphogenetic protein
BMSC: bone marrow stromal cell
EFS: event-free survival
DKK-1: Dickkopf 1
GEP: gene expression profiling
GPI: GEP-based proliferation index
HMCL: human myeloma cell line
IGF-1: insulin like growth factor 1
IGFBP7: Insulin like growth factor binding protein 7
MBC: memory B cell
MGUS: monoclonal gammopathy of undetermined significance
MM: multiple myeloma
MMSET: multiple myeloma SET domain
OS: overall survival
PC: plasma cell
PPC: polyclonal plasmablastic cell
TSA: trichostatin A
VEGF: vascular endothelial growth factor
