# Supplementary material for: Insulin like growth factor binding protein 7 (IGFBP7) expression is linked to poor prognosis but may protect from bone disease in multiple myeloma
Source: J Hematol Oncol. 2015 Feb 8;8:10. doi: 10.1186/s13045-014-0105-1 (PMC4333268; doi:10.1186/s13045-014-0105-1)
Supplement: Additional file 1: Table S1. — BMP antagonist expression in CD138+ purified cells. Bold indicates differential expression in MM compared to healthy donor bone marrow plasma cells (BMPCs). [file 13045_2014_105_MOESM1_ESM.doc]

| gene | Presence of expression | | | |
| --- | --- | --- | --- | --- |
|  | BMPC | MGUS | MM | HMCL |
| *TOB1* | 100 | 100 | 99.09 | 100 |
| *TWSG1* | 100 | 100 | 100 | 94.12 |
| ***IGFBP7*** | 100 | 45.45 | 47.72 | 47.06 |
| *SMURF2* | 90 | 90.91 | 96.66 | 100 |
| *SMAD7* | 90 | 86.36 | 80.55 | 94.12 |
| ***SKIL*** | 50 | 90.91 | 90.27 | 58.82 |
| *TSKU* | 50 | 27.27 | 25.84 | 58.82 |
| *SMURF1* | 30 | 36.36 | 37.39 | 76.47 |
| ***CHRDL1*** | 30 | 13.64 | 6.69 | 17.65 |
| *BAMBI* | 10 | 9.09 | 12.77 | 70.59 |
| *SMAD6* | 10 | 9.09 | 6.08 | 29.41 |
| *SKI* | 10 | 0 | 1.82 | 0 |
| ***FSTL1*** | 10 | 0 | 0.91 | 0 |
| *FST* | 10 | 0 | 2.13 | 0 |
| *SOSTDC1* | 0 | 0 | 0.30 | 5.89 |
| ***FSTL5*** | 0 | 13.64 | 21.88 | 23.53 |
| *FSTL3* | 0 | 0 | 0 | 5.88 |
| *DAND5* | 0 | 4.55 | 0 | 0 |
| *GREM2* | 0 | 0 | 0 | 5.88 |
| *GREM1* | 0 | 0 | 2.13 | 0 |

Supplementary Table S1. BMP antagonist expression in CD138+ purified cells. Bold indicates differential expression in MM compared to healthy donor bone marrow plasma cells (BMPCs).
