# Supplementary material for: Insulin like growth factor binding protein 7 (IGFBP7) expression is linked to poor prognosis but may protect from bone disease in multiple myeloma
Source: J Hematol Oncol. 2015 Feb 8;8:10. doi: 10.1186/s13045-014-0105-1 (PMC4333268; doi:10.1186/s13045-014-0105-1)
Supplement: Additional file 2: Table S2. — BMP antagonist expression pattern in whole bone marrow (WBM) samples. Bold indicates differential expression. [file 13045_2014_105_MOESM2_ESM.doc]

| gene | Presence of expression | |
| --- | --- | --- |
|  | ND-WBM | MM-WBM |
| *SMURF2* | 100 | 100 |
| *SMURF1* | 100 | 85.96 |
| *SMAD7* | 100 | 100 |
| *TOB1* | 100 | 100 |
| *SKIL* | 100 | 98.25 |
| *TWSG1* | 100 | 100 |
| ***IGFBP7*** | 100 | 100 |
| ***BAMBI*** | 85.71 | 42.11 |
| *SKI* | 14.29 | 15.79 |
| *FSTL3* | 14.29 | 3.51 |
| *FSTL1* | 14.29 | 21.05 |
| *SMAD6* | 0 | 8.77 |
| *TSKU* | 0 | 7.02 |
| *CHRDL1* | 0 | 7.02 |
| *NOG* | 0 | 1.75 |
| *FSTL5* | 0 | 10.53 |
| *FST* | 0 | 1.75 |
| *GREM1* | 0 | 1.75 |
| *NBL1* | 0 | 1.75 |

Supplementary Table S2. BMP antagonist expression pattern in whole bone marrow (WBM) samples. Bold indicates differential expression.
