# Supplementary figures and images for: Insulin like growth factor binding protein 7 (IGFBP7) expression is linked to poor prognosis but may protect from bone disease in multiple myeloma
Source: J Hematol Oncol. 2015 Feb 8;8:10. doi: 10.1186/s13045-014-0105-1 (PMC4333268; doi:10.1186/s13045-014-0105-1)

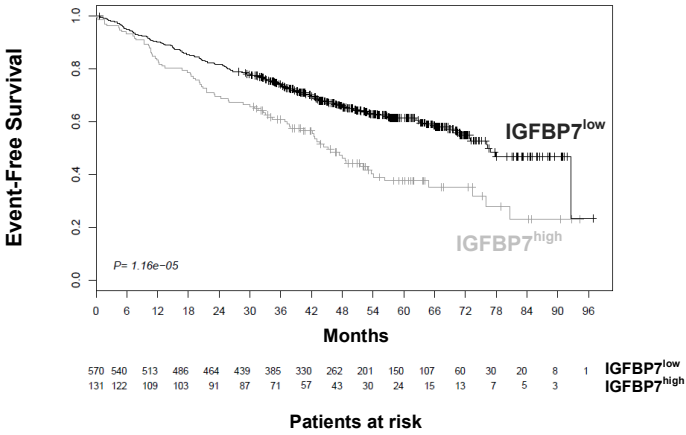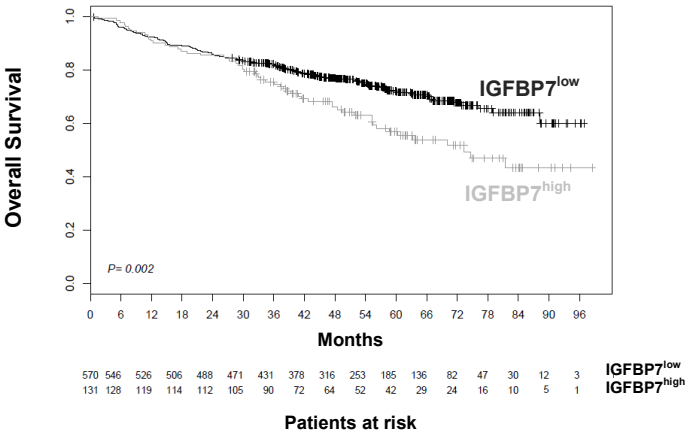

Supplement: Additional file 3: Figure S1. — EFS and OS in the LR cohort when the same fraction of patients defined as “high” IGFBP7 expressers in the HM-cohort (18.6%) was designated as high expressers in the LR-cohort. High IGFBP7 expression was associated with adverse event-free (P<0.001) and overall survival (P<0.002) by applying this criterion. [file 13045_2014_105_MOESM3_ESM.pdf]

**A**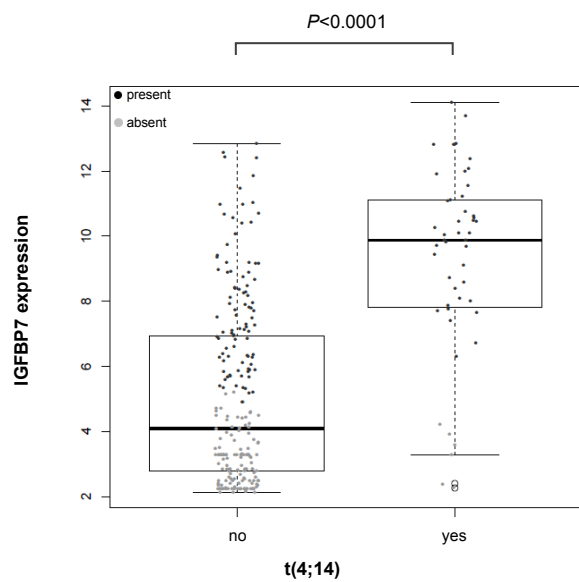**B**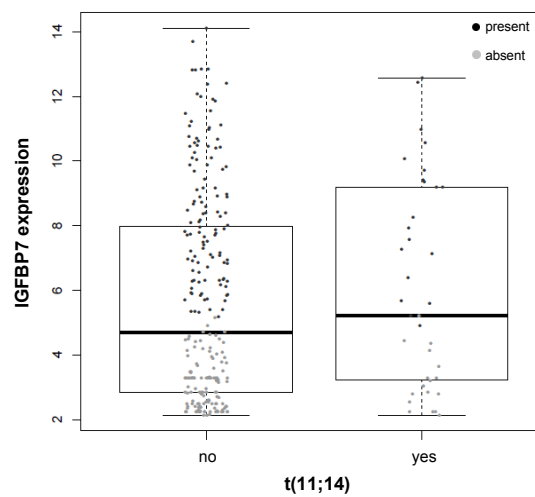**C**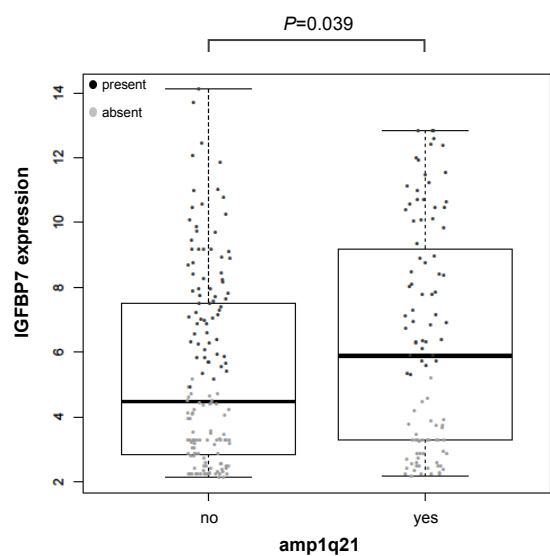**D**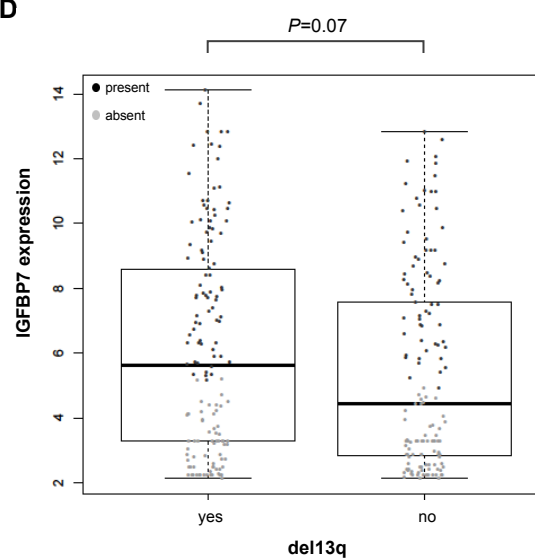**E**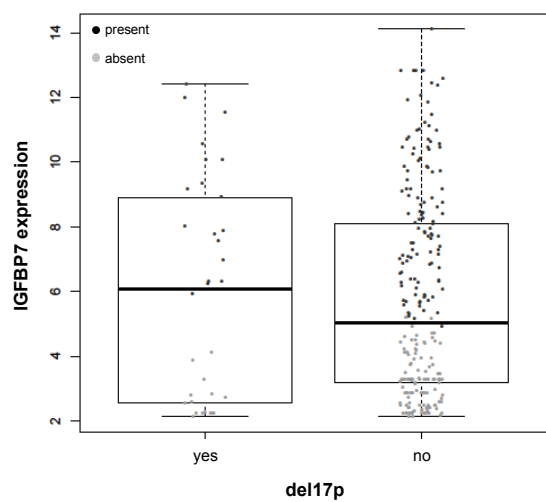

Supplement: Additional file 4: Figure S2. — IGFBP7 expression is associated with high risk cytogenetics. Gene expression analysis of the HM-patient cohort revealed that high IGFBP7 expression levels were associated with poor risk cytogenetic markers including (A) translocation t(4;14) and (C) amplification 1q21. Moreover, a trend was found regarding the more frequent presence of deletion 13q in patients with high IGFBP7 expression values (D). No association was observed for (B) translocation t(11;14) and (E) deletion 17p. Grey data points indicate an absent, black data points a present Affymetrix detection call. [file 13045_2014_105_MOESM4_ESM.pdf]

**A**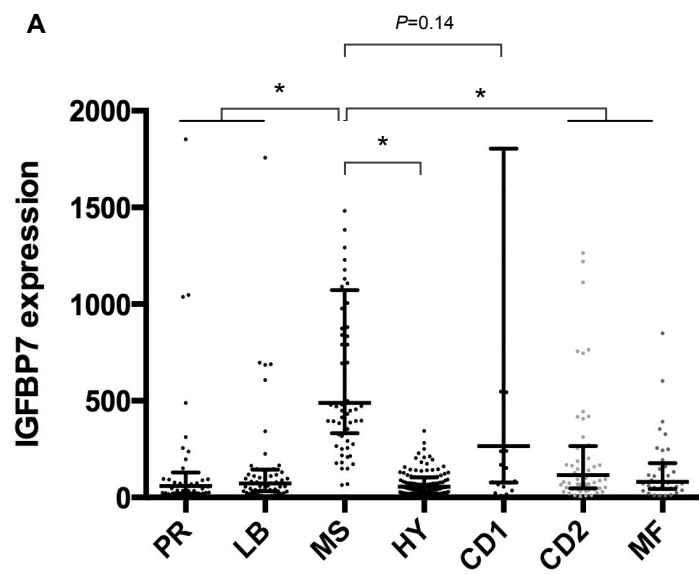**B**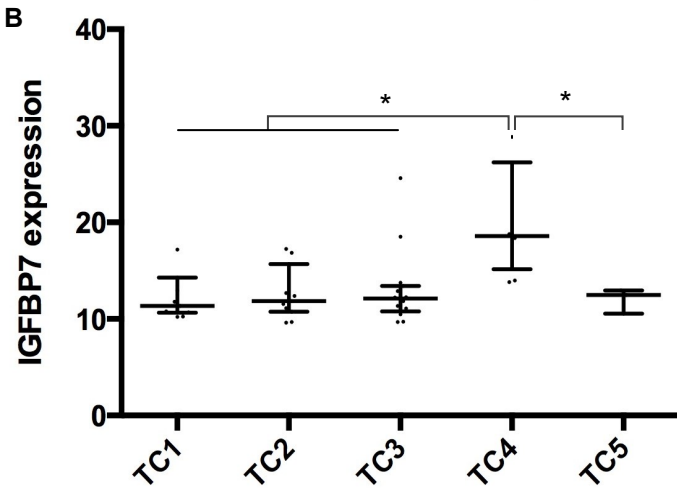

Supplement: Additional file 5: Figure S3. — IGFBP7 expression is elevated in MMSET defined molecular subgroups of myeloma. IGFBP7 expression was analysed in two publically available GEP datasets published by (A) Zhan et al. [33] and (B) Agnelli et al. [34]. Both datasets classified MM cases into distinct molecular subgropus based on microarray experiments. (A) IGFBP7 expression was significantly elevated in the MMSET (MS) overexpressing subgroup compared to five other GEP-defined subgroups. In addition, IGFBP7 expression tended to be elevated in the MS subgroup compared to the t(11;14) associated cyclin D1 (CD1) overexpressing samples. The other subgoups were defined by a proliferation (PR), low bone involvement (LB), hyperdiploid (HY), t(6;14) associated cyclin D (CD2) and MAF (MF) expression based GEP profile. (B) Analysis of the dataset from Agnelli et al. [34] confirmed overexpression of IGFBP7 in the MMSET defined molecular subgroup of MM (TC4). The other subgroups were defined by cyclin D overexpression (TC1), hyperdiploid status (TC2) and MAF overexpression (TC5.) TC3 was defined by the lack of association with any of the other subgroups. Asterisks indicate statistical significance compared with the other molecular subgroups (* P < 0.05). Horizontal lines represent median IGFBP7 expression with interquartile range. [file 13045_2014_105_MOESM5_ESM.pdf]
